# Supplementary material for: Influence of the Backward Fall Technique on the Transverse Linear Acceleration of the Head during the Fall
Source: Sensors (Basel). 2023 Mar 20;23(6):3276. doi: 10.3390/s23063276 (PMC10054543; doi:10.3390/s23063276)
Supplement: Supplementary file 1 [file sensors-23-03276-s001.zip › sensors-2173506-SI.pdf]

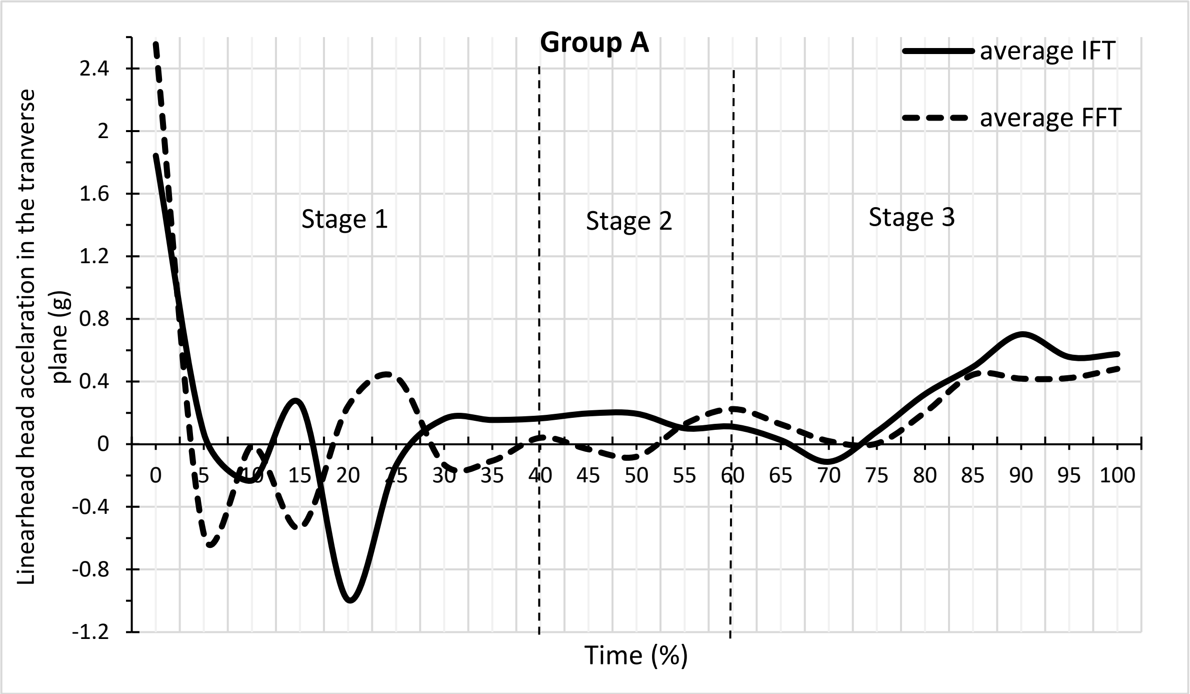

Figure S1. Dependence of mean values of linear head acceleration in the transverse plane on the time of performing a backward fall in the form of IFT and FFT in group A, which consisted of students training martial arts (from Table 1).

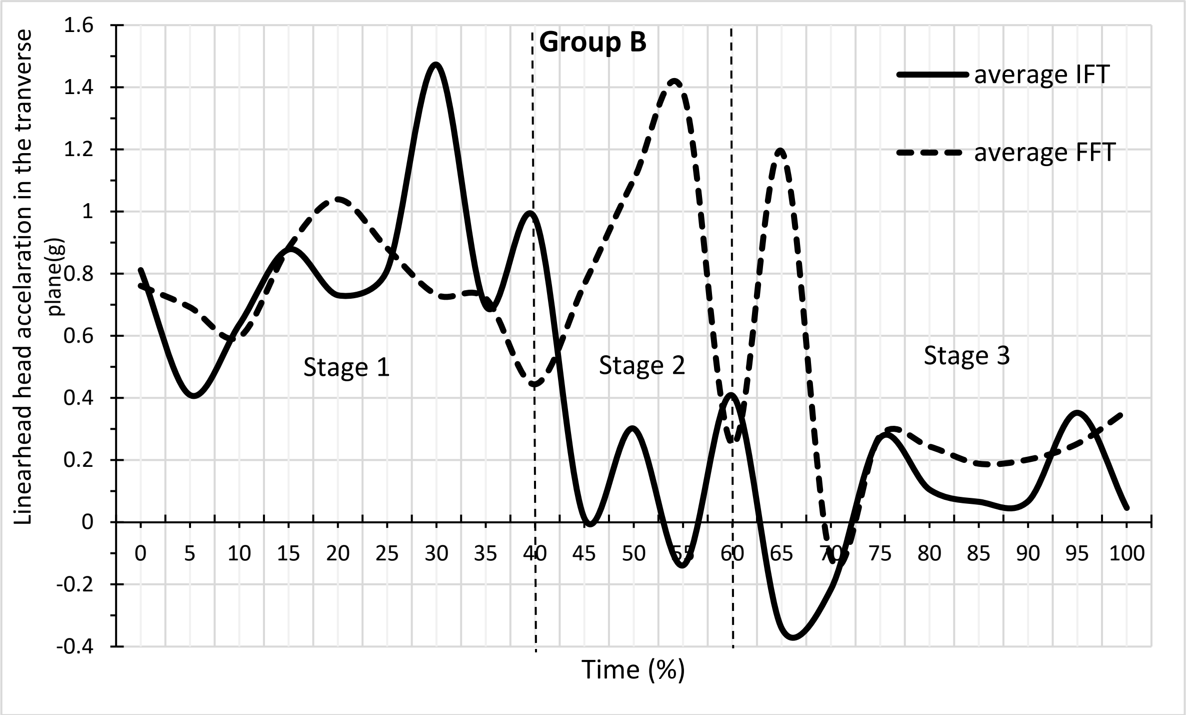

Figure S2. Dependence of mean values of linear head acceleration in the transverse plane on the time of performing a backward fall in the form of IFT and FFT in group B, which consisted of students who played handball (from Table 2).

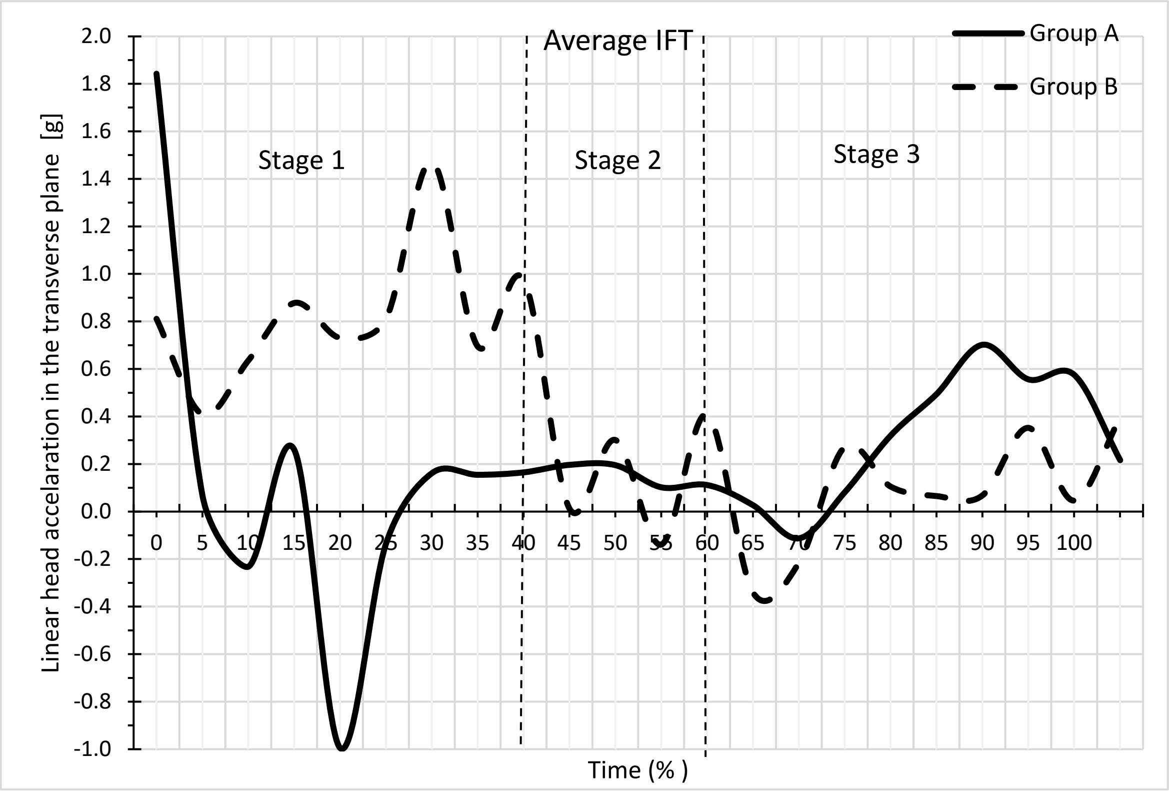

Figure S3. Dependence of mean values of linear head acceleration in the transverse plane at the time of performing a backward fall in the form of IFT between groups A and B (from Table 1 and Table 2).

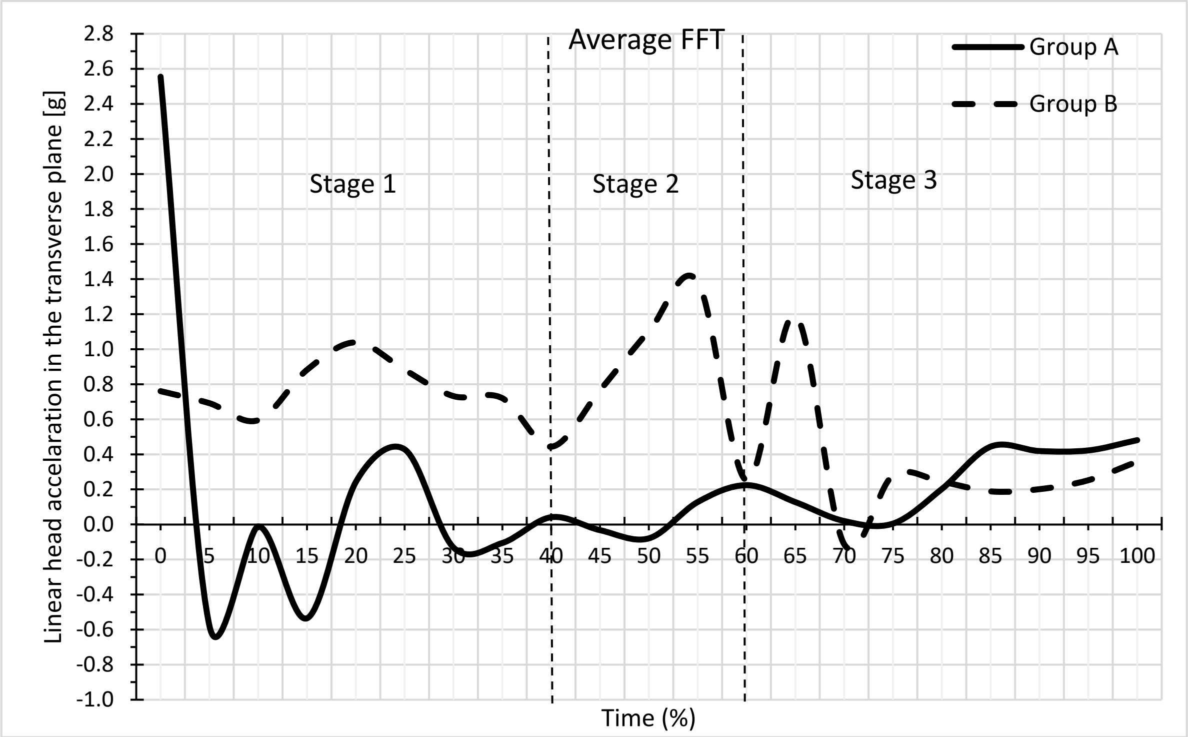

Figure S4. Dependence of mean values of linear head acceleration in the transverse plane at the time of performing a backward fall in the form of FFT between groups A and B (from Table 1 and Table 2).

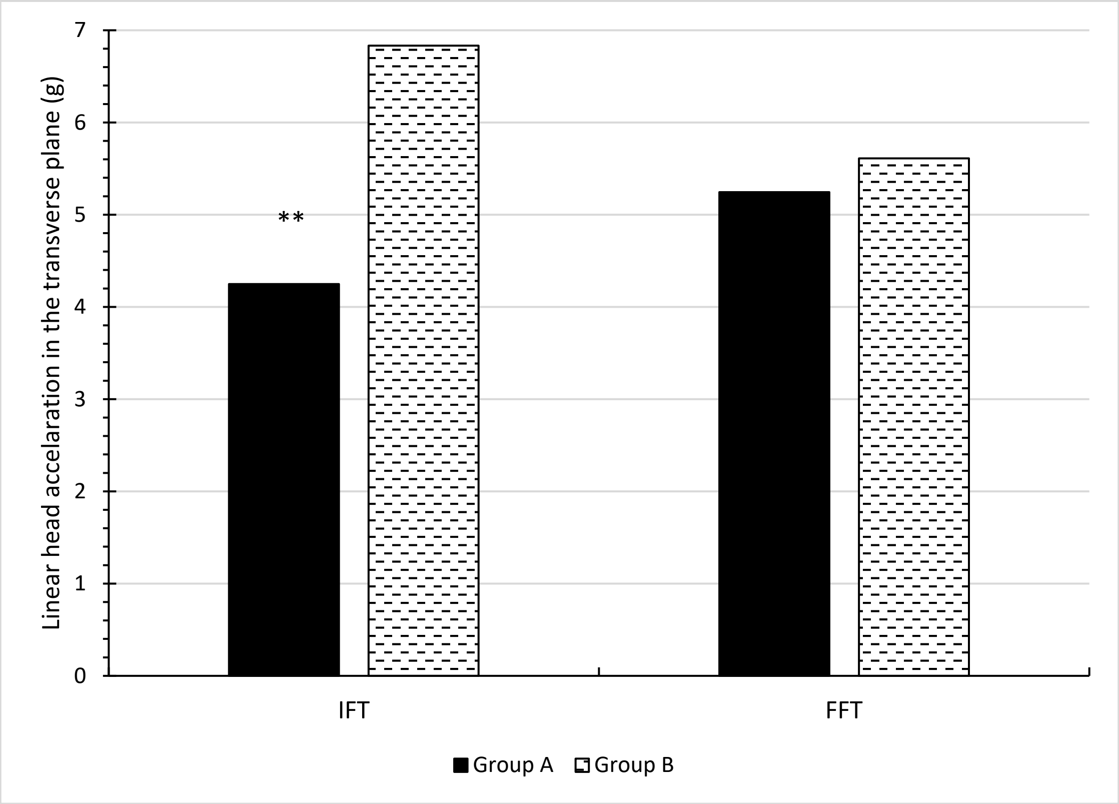

Figure S5. Comparison mean delta values for IFT and FFT between groups A and B (** means a significant difference between the mean values provided at the significance level 0.01 (from Table 6).

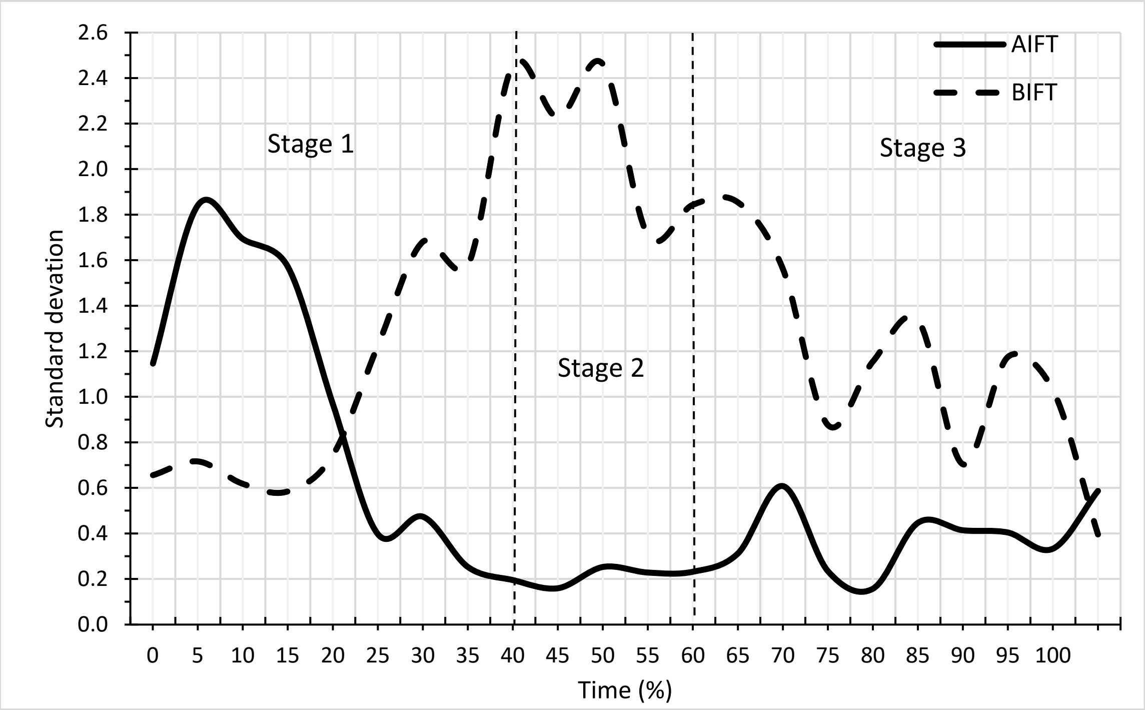

Figure S6. Dependence of the standard deviations on the time of performing a backward fall for FFT in groups A and B (from Table 7).

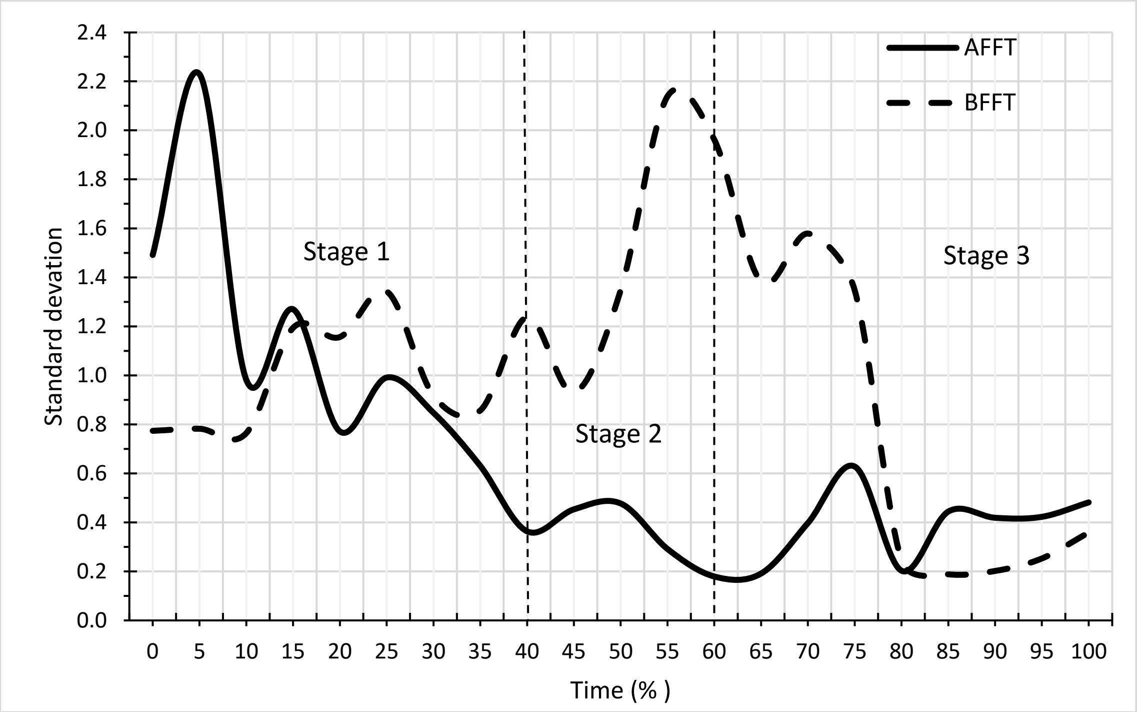

Figure S7. Dependence of the standard deviations on the time of performing a backward fall for FFT in groups A and B (from Table 8).
